# Inflammation Induced by Platelet-Activating Viperid Snake Venoms: Perspectives on Thromboinflammation

**DOI:** 10.3389/fimmu.2019.02082

**Published:** 2019-09-04

**Authors:** Catarina Teixeira, Cristina Maria Fernandes, Elbio Leiguez, Ana Marisa Chudzinski-Tavassi

**Affiliations:** ^1^Laboratory of Pharmacology, Butantan Institute, São Paulo, Brazil; ^2^Centre of Excellence in New Target Discovery, Butantan Institute, São Paulo, Brazil; ^3^Laboratory of Molecular Biology, Butantan Institute, São Paulo, Brazil

**Keywords:** snake envenoming, venom-induced inflammation, toxins, platelet activation, thromboinflammation

## Abstract

Envenomation by viperid snakes is characterized by systemic thrombotic syndrome and prominent local inflammation. To date, the mechanisms underlying inflammation and blood coagulation induced by *Viperidae* venoms have been viewed as distinct processes. However, studies on the mechanisms involved in these processes have revealed several factors and signaling molecules that simultaneously act in both the innate immune and hemostatic systems, suggesting an overlap between both systems during viper envenomation. Moreover, distinct classes of venom toxins involved in these effects have also been identified. However, the interplay between inflammation and hemostatic alterations, referred as to thromboinflammation, has never been addressed in the investigation of viper envenomation. Considering that platelets are important targets of viper snake venoms and are critical for the process of thromboinflammation, in this review, we summarize the inflammatory effects and mechanisms induced by viper snake venoms, particularly from the *Bothrops* genus, which strongly activate platelet functions and highlight selected venom components (metalloproteases and C-type lectins) that both stimulate platelet functions and exhibit pro-inflammatory activities, thus providing insights into the possible role(s) of thromboinflammation in viper envenomation.

## Introduction

### Thromboinflammation and Platelets

Coordinated activation of inflammation and hemostatic responses following tissue injury or invading pathogens is a defense mechanism with an essential role in homeostasis maintenance, leading to a thromboinflammatory response, elimination of pathogens, tissue remodeling, and repair (1). In general, thromboinflammation results from activation of the cascade systems of the blood (the complement, contact, coagulation, and fibrinolytic systems) and activation of a complex multicellular blood system comprising platelets, endothelial cells, and various subsets of inflammatory leukocytes. The functional interdependence among these systems has been largely described, and plentiful cross-talk occurring between different system cascades has been demonstrated (2–5). However, if not properly controlled, activation of these cascade systems will trigger severe thromboinflammatory responses, which can extend systemically and damage remote organs. In diverse pathological conditions, a pro-inflammatory state combined with thrombotic events can produce multiorgan failure as a consequence of excessive platelet activation, coagulation, and fibrin deposition in the microvasculature (5, 6).

Platelets are the predominant cellular elements in the process of thromboinflammation. These anucleate cells are critical in maintaining hemostasis and in arresting blood loss. Platelets contain three types of secretory granules that are essential to maintain steady state hemostasis: α-granules, the most abundant organelle in platelets, contain proteins endocytosed from plasma, or synthetized by megakaryocytes, such as PF4; dense granules contain nucleotides, serotonin, polyphosphate, calcium and magnesium; lysosomes contain acid hydrolases, such as β-hesosaminidase; and T granules. At the site of a vascular injury, platelets develop a series of highly regulated functions, which include adhesion to endothelial and subendothelial structures, followed by activation and aggregation, constituting the called first wave of hemostasis (7–9). Once platelets become aggregated, the coagulation cascade is initiated by the intrinsic or extrinsic cascades, leading to activation of prothrombin and culminating in thrombin generation. Thrombin, in turn, activates platelets by cleaving protease activated receptors (PARs) expressed on platelets' surface, leading to thrombus formation (10, 11). Thrombin is considered a central mediator of thromboinflammation and plays a central role in propagating microvascular thrombosis and inflammation (12). This mediator affects the vasculature components by cleaving components of coagulation, complement, and fibrinolytic systems, as well as by activating endothelial cells, leukocytes, vascular smooth cells, fibroblasts, and platelets (13–15).

A number of receptors are found on the membrane surface of platelets, such as GPIb-V-IX, GPVI, 5HT2A, TP, α2A, P2Y1, P2Y12, integrin receptors, PARs, and toll-like receptors (TLRs) (16–21). Engagement of these receptors by their agonists triggers functional activities of platelets, being essential for the roles played by these cells in both physiological and pathological processes (22, 23).

Beyond hemostasis and thrombosis, platelets are involved in diverse physiological and pathological processes, including the innate immune response. Yet platelets, like many host defense cells, can detect pathogen associated molecular patterns (PAMPs) via TLRs expressed on their membrane (24, 25), promoting thrombus formation. In response to PAMPs presented via TLR4/MYD88, platelets release microbicidal products (26) and induce neutrophil degranulation and release of neutrophil extracellular traps (NETs) (27). In addition to anti-bacterial functions, NETs display pro-coagulant and pro-thrombotic activities and bind to von Willebrand Factor (vWF), inducing platelet recruitment. Further, neutrophil-derived serine proteases and extracellular nucleosomes induce thrombosis and fibrin formation (28), strengthening coagulation through local proteolysis of tissue factor pathway inhibitor (TFPI). Moreover, platelet-derived PDI promotes the decryption/activation of tissue factor by monocytes, contributing to fibrin generation (29). Therefore, platelets are immune effector cells since they are able to both recognize pathogens via TLRs and communicate with innate immune cells to enhance their prothrombotic functions. Moreover, they act as contact elements between the complement, coagulation, and contact activation systems (30). In this context, a variety of innate and adaptive immune responses and hemostatic disturbances are induced by animal venoms.

### Snake Venom

Snake venoms consist of a complex mixture of bioactive molecules, known as toxins, which are delivered in their victims through bites and used for defense or predation. Toxins in turn exhibit a great diversity of chemical composition, including proteins, peptides, biogenic amines, lipids and polysaccharides, and exhibit strong activity and high specificity for their targets, which comprise virtually all physiological systems and tissues.

Venomous snakes belong to four main families, *Viperidae, Elapidae, Atractaspididae*, and *Colubridae*, and their venoms contain substances with diverse biochemical and biological activities. The *Viperidae* family comprises a medically important group of venomous snakes and accounts for the majority of death and morbidity worldwide compared to the others (31, 32). Snakes of this cosmopolitan family are currently arranged in three subfamilies: *Viperinae, Azemiopinae*, and *Crotalinae* (33). The *Crotalinae*, or “pit vipers,” subfamily is the most diverse and widely distributed lineage of vipers and comprises species occurring both in the Old and New World (34). In general, viper snakebite envenomation causes systemic and prominent local effects in the victims. Systemically, these venoms induce hemostatic disturbances related to consumption coagulopathy via the action of their pro-coagulant toxins and bleeding, which may evolve into cardiovascular and respiratory failure and death (35, 36). Local effects include inflammation, with edema, pain and conspicuous leukocyte infiltration, hemorrhage, and myonecrosis.

Regarding systemic effects, there are clinical reports describing systemic and venous thrombosis and multiple cerebral, myocardial, and mesenteric infarctions in victims envenomed by *Bothrops* sp. snakes. In the case of envenomation induced by *B. lanceolatus*, histopathologic examination of cerebral, myocardial, and mesenteric small arteries and arterioles showed multifocal thrombotic microangiopathy and platelet aggregates, being endothelial cells found within microthrombi (37, 38).

Coagulopathy induced by viperid snake venoms is a consequence of activation of the clotting pathway by pro-coagulant toxins present in these venoms, resulting in clotting factor consumption. The enzymatic toxins interfering with coagulation are pro-coagulant proteases (prothrombin activators, thrombin-like enzymes, factor X, and factor V activators) and anticoagulant proteases (factor IX and X inhibitors, protein C activator, anticoagulant PLA_2_s). The venom components acting on fibrinolysis are fibrinolytic enzymes and plasminogen activators (39–42). Finally, viperid venoms are known to act on platelets, and several venom components show high structural and functional similarities to different natural ligands of the platelet adhesion receptor, thus affecting platelet functions by different mechanisms, including (i) binding or degradation of vWF or platelet receptors, e.g., α2β3 integrin, (ii) activation of protease-activated receptors (PARs) by thrombin-like enzymes, (iii) modulation of adenosine diphosphate (ADP) release, and (iv) modulation of thromboxane A_2_ (TXA_2_) formation. Both inhibition and activation of platelets by venom components contribute to venom-induced coagulopathy by depleting platelets, culminating in marked thrombocytopenia (39, 43–45).

Regarding activation of platelets, although the effects of isolated venom toxins have been investigated in detail, the potent activity of whole venoms of several species of *Viperidae* snakes to stimulate platelet function has not been well-established. *B. jararaca* snake venom has been demonstrated to activate platelet PARs, leading to platelet aggregation through the action of thrombin-like enzymes and prothrombin activators (46, 47). In addition, pro-secreting activity of *B. jararaca* on mouse and human washed platelets has been recently described. Release of PF4 and β-hexosaminidase from platelet α-granules and lysosomes, respectively has been demonstrated (45). Increased expression of TF at the site of *B. jararaca* venom injection and in lungs of rats, as well as decryption of this factor, were found in an experimental model of envenomation. In this condition, levels of TF were increased in plasma, indicating that disseminated intravascular coagulation syndrome may occur during *Bothrops* envenomation (48). Similarly, *B. atrox*, and *B. asper* snake venoms are also able to activate platelet PARs by virtue of their thrombin-like enzymes (49, 50). In the case of *B. asper*, the presence of pro-thrombin activators is also evidenced (51). In addition, *Crotalus durissus* snake venoms and those from diverse species of *Trimeresurus* genus have also been demonstrated to activate platelets via the action of their thrombin-like enzyme content (49, 52–54).

## Inflammation Induced by Viper Snake Venom

Inflammation is largely recognized to be closely associated with the onset of local and systemic toxicity induced by *Crotalinae* venoms. Although still incomplete, studies on the inflammatory activities of snake venoms in the *Crotalinae* subfamily, mainly those of the *Bothrops* genus, are robust in comparison with others of the same family and subfamily. Therefore, in this review, emphasis will be placed on the present knowledge of inflammatory reactions induced by *Bothrops* sp. snake venoms.

*Bothrops* sp. snake venom induces a set of gross inflammatory events, including edema, leukocyte migration, and a complex network of released mediators. These events can progress to either resolution or an excessive and uncontrolled inflammatory response, depending on the volume of venom injected into the victim.

Severe local edema is a relevant clinical finding in *Bothrops* snake-bitten victims (55, 56) that frequently leads to ischemia and neural compression, contributing to tissue loss and disability (57–59). Experimental studies on the dynamics of local inflammation caused by *B. asper* venom have demonstrated early plasma extravasation from small venules and adjoining capillary segments a few minutes after exposure to venom, followed by stasis in the microcirculation (60). The edematogenic response to *Bothrops* venoms was shown to be dependent on local release of well-known synergistic inflammatory mediators, which cause increased vascular permeability and/or vasodilation. Accordingly, the roles of histamine via H1 and H3/4 receptors, serotonin, bradykinin (61–63), platelet activating factor (PAF) (64), prostaglandins (PGs) derived from both cyclooxygenase 1 and 2 pathways (64–66), neurokinins (67), nitric oxide (NO) (68), and α-1 and α-2 adrenergic mediators (61, 69) have been demonstrated in several experimental studies. Participation of anaphylatoxins, including the complement components C3 and C5, in edema formation has also been shown (70–74). The diversity of mediators participating in edema formation induced by *Bothrops* sp. venoms can be partially explained by the ability of these venoms to induce degranulation of mast cells (62, 63). Furthermore, the ability of *Bothrops asper* snake venom to contract collecting lymphatic vessels of mouse mesentery followed by halting the flow of lymph was demonstrated, suggesting that disturbance of the lymphatic system by venom may contribute to development of edema in envenomed tissues (75).

A conspicuous infiltration of leukocytes into the local snakebite is another important characteristic of inflammation triggered by the majority of *Viperidae* viperid snake venoms (55, 56). Leukocytes are essential cells in host defense, and they respond to injury by releasing inflammatory mediators, performing phagocytosis and inducing production of potent microbicidal products. Several experimental studies have demonstrated that *Bothrops* sp. venoms induce time-dependent infiltration of leukocytes into the site of the bite, characterized by an early neutrophil influx followed by a late mononuclear cell infiltrate, that accumulates at the site of venom injection and in adjacent tissues (66, 76–79). Moreover, studies on the mechanisms of *Bothrops* snake venom-induced leukocyte recruitment have supported species-dependent differences in the ability of these venoms to activate the intrinsic chemotactic activities of neutrophils. Whereas, *B. jararaca* neither triggers oriented cell locomotion nor modifies the intrinsic ability of neutrophils to migrate in response to a chemoattractant factor (80), *B. jararacussu* induces neutrophil chemotaxis *in vitro* by a direct mechanism (81). Such variability is explained by the presence of a lectin in *B. jararacussu* venom that recognizes glycoligands on neutrophil surfaces, leading to chemotaxis of neutrophils (82, 83). Moreover, the ability of *Bothrops* sp. snake venom to stimulate expression of the adhesion molecules L-selectin, LFA-1, ICAM- 1, PECAM-1, and CD18 was described in an *in vivo* experimental model in mice (84). This effect was related to the action of inflammatory cytokines and leukotrienes released by the venom acting on endothelial cells and/or leukocytes (84–86).

Cytokines and chemokines are important mediators of cell-cell communication and major mediators of the upregulation of adhesion molecule expression and chemotaxis of leukocytes (87). The involvement of cytokines and chemokines in *Bothrops* snake venom-induced recruitment of leukocytes has been suggested by many to be due to increased levels of Th1 class cytokines, the regulatory cytokine IL-10 and chemokines found at the site of injection of distinct species of *Bothrops* snake venoms (64, 66, 77, 86, 88–92). Moreover, increased serum levels of IL-1β, IL-6, TNF-α, IL-10, and IFN-γ were found after injection of whole venoms from *B. jararaca* and *B. asper* into mice (93). Accordingly, increased levels of IL-6, IL- 8, TNF-α, MIP-1α, and RANTES were detected in the serum of patients bitten by *B. jararaca* and *B. asper* snakes (94). In addition, the direct action of venoms on leukocytes, primarily neutrophils, and macrophages, lead to production and release of mediators. Release of COX1- and COX2- derived prostaglandins, including PGE_2_, PGD_2_, and TXA_2_ and the 5-LO-derived LTB_4_, by neutrophils and macrophages has also been described (85, 86, 94–96). Further, formation of NETs upon stimulation by distinct *Bothrops* sp. venoms was also recently demonstrated (97).

A recent study demonstrated that *B. jararaca* and *B. jararacussu* venoms induce nuclear translocation of the transcription factor NF-κB in human monocytes and mouse alveolar macrophages, and this transcription factor participates in production of IL-1β. In addition, venom-induced activation of NF-κB was shown to be upregulated by PGE_2_ but downregulated by LTB_4_ (90). Although cytokines and eicosanoids are relevant for driving inflammatory events, the regulatory mechanisms underlying production of each group of mediators by *Bothrops* venoms and the cross-talk occurring between mediators has yet to be further investigated. Moreover, despite the importance of inflammasome assembly in the development of inflammatory processes (98), the potential role of this multiprotein complex in the inflammatory responses to snake venoms is still poorly understood. The beneficial role of the inflammasome in the host response to *Crotalus atrox* was demonstrated early (99), arguing for contribution of the inflammasome platform to the inflammatory effects of viper snake venoms.

In addition to the involvement of diverse inflammatory mediators, leukocyte infiltration induced by *Bothrops* snake venoms has been associated with generation of chemotactic fractions from the complement system. Participation of the C5a fraction of the complement system in *B. asper* venom-induced leukocyte migration was demonstrated in C5-deficient animals (100). The ability of several species of *Bothrops* snake venoms to activate the complement system, generating chemotactic fractions, has been demonstrated (70, 72, 74). In this regard, activation of the complement cascade by 19 different *Bothrops* species from South and Central America was demonstrated to occur by one or more pathways through their action either by directly cleaving C3 and C5 factors or by inactivating the soluble C1 inhibitor, C1-INH factor (71, 72). Moreover, activation of the complement system by *B. lanceolatus* snake venom in human serum has been reported (101).

Another important feature of *Bothrops* sp. venoms is related to their ability to stimulate leukocyte functions, primarily production of microbicidal substances and phagocytosis. Studies have showed generation of reactive oxygen species (ROS) by macrophages and peritoneal leukocytes stimulated with *Bothrops* sp. snake venoms (97, 102). Further, increased production of hydrogen peroxide and nitric oxide by macrophages followed by generation of peroxynitrites induced by *Bothrops* snake venoms (103) and the ability of *Vipera russelli*, a snake of medical importance in Asia, to activate production of ROS in neutrophils were also described. Prolonged or excessive release of leukocyte-derived peroxynitrites and ROS enhances inflammatory processes and potentiates tissue damage at inflammatory sites (104–106). Studies attempting to block the damaging effects of either *B. asper* or *Vipera russelli* snake venoms using different antioxidant compounds and/or inhibitors of NO production did not show similar protective effects (105, 107). Interestingly, the beneficial role of neutrophils in host defense against the noxious effects of snake venoms was demonstrated by studies showing that experimentally induced neutrophil depletion in mice reduced skeletal muscle regeneration after myonecrosis induced by *B. asper* snake venom and a myotoxic phospholipase A_2_ isolated from this venom. Absence of these phagocytic cells impaired the late recruitment of macrophages into the damaged tissue, abolishing the clearance of necrotic *debris* (108). In this sense, the role of neutrophils in regulating production of NO and some cytokines and chemokines was further demonstrated in mice injected with *B. atrox* (88).

A number of surface and cytosolic receptors expressed in innate immune cells are able to sense PAMPs, damage-associated molecular patterns (DAMPs), or venom-associated molecular patterns (VAMPs) with high sensitivity and specificity, namely, TLRs (109, 110). Once engaged, these recognition receptors trigger signaling pathways that culminate in the transcription of inflammatory genes related to inflammatory mediators, including cytokines and eicosanoids. Adaptor proteins, such as the myeloid differentiation factor 88 (MyD88), mediate major TLR-activated intracellular signaling pathways (110, 111). Although the importance of TLRs in triggering inflammation is well-recognized, studies on their participation in snake venom-induced inflammation are scarce. Participation of TLR2 in inflammation via the MyD88 adaptor molecule induced by *B. atrox* snake venom was demonstrated in MyD88 knockout mice. These animals showed low infiltration of leukocytes into the site of venom injection and failed to produce Th1 and Th17 cytokines, CCL-2, PGE_2_, and LTB_4_ (112, 113). Moreover, TLR2 was shown to be involved in the release of PGE2, IL-1β, and IL-10 by macrophages stimulated with phospholipase A_2_ (MT-III) isolated from *B. asper* snake venom (114). Consistent with the importance of inflammation in tissue regeneration, the involvement of TLR4 in protection against muscular damage induced by *B. jararacussu* snake venom in mice has been reported (115). Moreover, besides inflammatory mediators, the exudate generated in *Bothrops* venom-damaged tissue was shown to contain a large array of DAMPs derived from the affected cells and extracellular matrix (92). Among the DAMPs identified in exudates of mice injected with *B. asper* venom, several are known to play roles in the inflammatory reaction, coagulation and complement systems and may be critical elements for the overlap between inflammation and thrombosis, thus favoring thromboinflammation. For instance, among identified DAMPs is fibrinogen, a component of the coagulation system with the ability to stimulate chemokine secretion by macrophages through TLR4 engagement (116), potentially favoring thromboinflammation. Furthermore, considering the presence of TLRs on platelets' surface and formation of DAMPS induced by viper venoms, it is plausible to suggest that thromboinflammation plays a role in the pathogenesis of viper envenomation. Hence, snake venom generates DAMPs by inducing inflammatory events and at the same time stimulating platelets and/or activating coagulation factors or endothelial cells, which may set up a pathway of thromboinflammation. This may thus culminate into formation of microthrombi, which may be critically associated to pathogenesis of snake envenomation.

### Inflammation Induced by Snake Venom Toxins

To date, studies reporting venom components endowed with the ability to reproduce both the inflammatory and platelet activating effects induced by the whole venom are few. Among the best studied toxins are metalloproteinases and C-type lectin like proteins.

### Snake Venom Metalloproteases

Coagulopathy following snake envenomation is triggered by pro-coagulant isolated toxins, in which metalloproteases play a relevant role by activating platelet function, the coagulation cascade, and fibrinolysis (48, 117–122).

The snake venom metalloproteases (SVMPs) comprise a subfamily of zinc-dependent enzymes of varying molecular mass and can be divided into three classes, depending on their domain organization: P-I, comprising only the metalloproteinase domain; P-II, having a metalloproteinase domain followed by a disintegrin-like domain; and P-III, comprising metalloproteinase, disintegrin-like, cysteine-rich domains and a lectin-like domain linked by disulfide bonds (123). Literature indicates that the precursor of SVMPs can be proteolytically cleaved at various interdomain sites, yielding molecules containing only the disintegrin or only the catalytic domain, followed by the disintegrin domain or even the complete SVMP containing all domains. Thus, the same venom may contain SVMPs with different molecular weights and variable domains (123, 124).

SVMPs have been reported to induce an intense inflammatory response, which has been associated with inflammation induced by the whole venom of *Bothrops* sp. snakes. In this context, Zychar et al. (125) demonstrated that the intensity of edema and hyperalgesia/allodynia, along with the interaction of leukocytes with endothelium, were significantly reduced in animals injected with *B. jararaca* snake venom pretreated with an inhibitor of SVMPs, confirming these enzymes as relevant contributors to the inflammatory reaction seen during *Bothrops* envenomation.

*In vivo* studies have demonstrated that SVMPs *per se* are able to induce increased levels of pro-inflammatory mediators and migration of leukocytes at the site of injection. After intraperitoneal injection, BaP1, a PI class SVMP from *B. asper* venom, induces increased levels of circulating neutrophils, followed by their infiltration into the site of injection. Participation of LECAM-1, CD18 and LFA-1 adhesion molecules in this effect was demonstrated. Moreover, increased levels of IL-1β and TNF-α in peritoneal washes collected from mice injected with BaP1 was shown (126). In addition, neuwiedase, another PI class SVMP from *B. neuwiedi* snake venom, was shown to induce inflammatory infiltration into gastrocnemius muscle and release of inflammatory mediators, such as KC, IL-1β, and IL-6, after intramuscular injection (127). Recently, the ability of the PI class BpirMP SVMP from *B. pirajai* snake venom to induce paw edema, recruitment of leukocytes and increased levels of nitric oxide, IL-6 and TNF-α in the peritoneal exudate has been demonstrated. Furthermore, these events were shown to be mediated by mast cell degranulation, histamine, prostaglandins, and cytokine production (128). Regarding PIII class SVMPs, the capability of jararhagin, isolated from *B. jararaca* snake venom, to induce infiltration of leukocytes into the mouse air pouch, dependent on activation of macrophages and the proteolytic activity of this SVMP, has been demonstrated (129).

The ability of SVMPs to activate different cell types and trigger pro-inflammatory responses *in vitro* has also been evidenced. Moojenactivase (MooA), a PIII class SVMP from *B. moojeni* snake venom, has been shown to stimulate production of the inflammatory mediators TNF-α, IL-8, and MCP-1 by peripheral mononuclear cells (122). After stimulation with neuwiedase, murine peritoneal adherent macrophages release significant levels of pro-inflammatory mediators, such as KC, IL-1β, and IL-6. Stimulation of satellite muscle cells (lineage C2C12) with neuwiedase caused release of the KC chemokine (127). In addition, HF3, a PIII class SVMP isolated from *B. jararaca* venom, induced phagocytosis of opsonized zymosan particles by eliciting macrophages in a process dependent on engagement of the αmβ2 integrin (130). Another PIII class SVMP, jararhagin, was shown to stimulate production of inflammatory mediators by murine macrophages, increasing mRNA translation for IL-6, TNF, and IL-1 (131). This SVMP also up-regulated gene expression of IL-8, IL-11, CXCL2, IL-1β, IL-6, and mammalian matrix metalloproteases (MMPs)-1 and 10 (132). Regarding activities on endothelial cells, berythractivase, a non-hemorrhagic PIII class SVMP, has been reported to induce an inflammatory state in these cells by upregulating expression of adhesion molecules, such as ICAM-1 and E-selectin, and inducing release of IL-8, NO, and vWF (133).

In addition, the ability of BaP1 to activate classic and alternative complement pathways, generating the chemotactic C5a factor, has been demonstrated. Accordingly, BaP1-treated serum developed the ability to induce chemotaxis of neutrophils *in vitro* (100). In line with these findings, participation of the complement system in the migration of inflammatory leukocytes into the site of injection of PI class SVMP from *B. atrox* snake venom has also been demonstrated (70).

In addition to pro-inflammatory activities, SVMPs can activate platelet functions by diverse mechanisms, including activation of the coagulation cascade, by inducing activation of prothrombin, vWF, factor X, and II and the complement cascade (C5a and C3a release), as well as engagement of platelet glycoprotein receptors. Prothrombin activating SVMPs are classified into A-D groups of prothrombin activators with the ability to cleave prothrombin, generating meizothrombin, which is then converted into α-thrombin, a potent mediator of microvascular thrombosis and inflammation (134–136). The first group of these factors comprise SVMPs isolated from *Bothrops* snake venoms of both PI and PIII classes. Examples include bothrojaractivase, isolated from *B. jararaca* snake venom (120), insularinase A from *B. insularis* (119), cotiarinase from *B. cotiara* (121), and berythractivase, isolated from *B. erythromelas* (118). Of note, besides inducing release of vWF, berythractivase has also been demonstrated to upregulate tissue factor (TF) expression in endothelial cells *in vitro*, displaying potent systemic thrombogenic activity, which in association with a generation of thrombin and inflammatory mediators, may contribute to thromboinflammation by favoring formation of systemic microthrombi (137).

SVMPs that are able to activate factor X have also been isolated from viper snake venoms and encompass P-III class enzymes (138). Factors II and X, found in the plasma as zymogens, can be activated by SVMPs via proteolytic cleavage of specific peptide bond sites, resulting in blood clotting (139). In this sense, the SVMP moojenactivase (MooA) induces human plasma clotting *in vitro* by activating coagulation factors II (prothrombin) and X, which in turn generate α-thrombin and factor Xa, respectively. Additionally, MooA induces aggregation of washed platelets and expression of TF on the membrane surface of peripheral blood mononuclear cells (122).Tissue factor has been reported to be involved in both thrombosis and inflammation, and several mediators, including pro-inflammatory cytokines and thrombin, induce its expression (140, 141). In this context, MooA is another promising candidate as an inducer of thromboinflammation during *Bothrops* envenomation.

Platelet adhesion and aggregation may be initiated by engaging specific membrane receptors that lead to platelet activation. Botrocetin, an SVMP isolated from *B. jararaca* snake venom, is an example of a potent activator of platelet function because it induces vWF- and platelet glycoprotein Ib (GPIb)-dependent platelet agglutination *in vitro*, resulting in platelet agglutination. Botrocetin appears to act in a two-step manner; first, it binds to vWF, forming a complex, which then binds to GPIb, causing platelet agglutination (142, 143). Moreover, other SVMPs, such as jararhagin, a PIII class SVMP, jaracetin, a differentially processed form of jararhagin lacking the catalytic domain, and the one-chain botrocetin, which corresponds to the disintegrin and cysteine-rich domains of jararhagin from *B. jararaca* snake venom, are able to activate vWF and its binding to platelet GP Ib-IX-V (144, 145). Interestingly, both jararhagin and jaracetin, but not botrocetin, are able to block the adhesion of collagen to platelet integrin α2β1 (145). These data demonstrate that SVMPs of the same class can activate different molecular targets to induce their actions on platelets.

The diversity and abundance of SVMPs in *Viperidae* snake venoms and their ability to induce inflammatory and pro coagulation events make them one the most promising toxin families for studies related to the characterization of venom-induced thromboinflammation.

### Snake Venom C-type Lectins

The C-type lectin family comprises proteins that recognize and bind carbohydrates in a Ca^2+^-dependent manner (146, 147) and non-sugar-binding snake venom C-type lectin-related proteins (SV-CLRPs), also called snaclecs (148, 149). These proteins exhibit the C-type lectin domain (CTLD) but differ from it in a long loop that either contributes to the sugar-binding site or is expanded into a loop-swapping heterodimerization domain between two CLRP subunits. SV-CLRPs connect with a multitude of molecules implicated in hemostasis that are present on endothelial cells, coagulation factors, and receptors of platelets, having a role in thrombus formation and inflammation (150).

With regard to inflammation, interaction of SV-CLRPs with immune cells, such as peripheral mononuclear cells and neutrophils, has been shown with the galactose-binding lectins from the venoms of *B. jararacussu* and *B. leucurus* snakes (82, 151). In addition to inducing edema and increasing vascular permeability in murine experimental models (152), BJcul from *B. jararacussu* venom has been demonstrated to stimulate phagocytosis of zymozan particles by macrophages and increase the lysosomal volume in neutrophils. In addition, BJcuL delays apoptosis of neutrophils and stimulates peripheral mononuclear cells to produce superoxide anions and hydrogen peroxide (82, 151). In human monocytes, BJcul up-regulates expression of antigen presentation molecules and enhances TNF-α, GM-CSF, and IL-6 synthesis by macrophages. Under inflammatory conditions, BjcuL induces macrophages into the M1 state of functional activation and indirectly stimulates T cells to produce TNF-α, IFN-γ, and IL-6 in the presence of LPS (151, 153). Moreover, galatrox, a C-type lectin from *B. atrox* snake venom, has been reported to interact with LacNAc-bearing glycans on neutrophils and macrophages, as well as with extracellular matrix proteins, leading to the release of pro-inflammatory mediators, such as IL-6, TNF-α, and keratinocyte-derived chemokine (KC). In an *in vivo* experimental model, galatrox induced marked neutrophil migration and was shown to induce release of pro-inflammatory cytokines IL-1α and IL-6 in the peritoneal cavity of mice. These effects were mediated by activation of the TLR4-MyD88 signaling pathway by the galatrox carbohydrate domain (154).

Homeostasis of blood coagulation depends on platelet activation, a process closely linked to inflammation (4). In this context, SV-CLRPs have been shown a broad and partially overlap with the platelet receptor-binding spectrum (155–157). Activation of platelets by SV-CLRPs occurs in a non-enzymatic manner, leading to inadequate thrombus formation and vessel occlusion. Conversely, some SV-CLRPs inhibit binding of the physiological ligands, antagonistically preventing the receptor from eliciting signals and resulting in severe bleeding. SV-CLRPs reported to agglutinate platelets via binding to GPIb include agglucetin from *Agkistrodon acutus* venom (158, 159), alboaggregin-B from the *Viperidae* venom *Trimeresurus albolabris* (160–162), mucrocetin and mucetin from *Trimeresurus mucrosquamatus* (163, 164), and jerdonuxin from *Trimeresurus/Protobothrops jerdonii* (164, 165). Agglucetin and alboaggregin-B also bind vWF receptor, but neither increase intracellular Ca^2+^ ions nor trigger platelet degranulation (158, 159). This suggests that platelets can be cross-linked and agglutinated by SV-CLRP-mediated GPIb multimerization, whereas a physiological agonist (vWF) elicits an active signaling process. Stejnulxin from *Trimeresurus stejnegeri* venom has been reported to activate platelets via GPVI (156, 166), a receptor for collagen on the platelet surface. However, EMS16 from *Echis multisquamatus* (166–169) binds to the collagen receptor of platelets, integrin α2β1, inhibiting the binding of collagen (170–172). Of note, α2β1 has been hypothesized to be responsible for strong adhesion of platelets to collagen, an event necessary for thrombus formation (172).

A platelet activation receptor, C-type lectin-like receptor 2 (CLEC-2), has been recognized as a platelet receptor activated by proteins isolated from snake venoms as evidenced by rhodocytin/aggretin (SV-CLRPs) from *Calloselasma rhodostoma* venom (173). CLEC-2 is expressed in immunocompetent cells, such as dendritic cells, monocytes, and neutrophils, and strongly expressed in platelets/megakaryocytes (174, 175). This receptor triggers strong activation of platelets through the tyrosine kinase dependent pathway. CLEC-2 has a single YxxL motif in its cytoplasmic tail, called hemi-ITAM because it resembles ITAM (tyrosine-based activation motif, YxxL-[X]10-12-YxxL), which has two YxxL motifs. ITAM is a signaling motif found in immune receptors, such as the T-cell receptor and the platelet collagen receptor GPVI–FcRc-chain complex. Engagement of CLEC-2 or GPVI triggers a signaling cascade culminating in platelet activation/aggregation (176) and thrombus formation (172, 177, 178).

Together, the above information evidences the high selectivity of various SV-CLRPs to platelet receptors, which are involved in activation of both platelet aggregation and inflammatory cascades, such as vWF and collagen receptor, known to be generated upon tissue injury.

## Concluding Remarks

**Figure 1:**
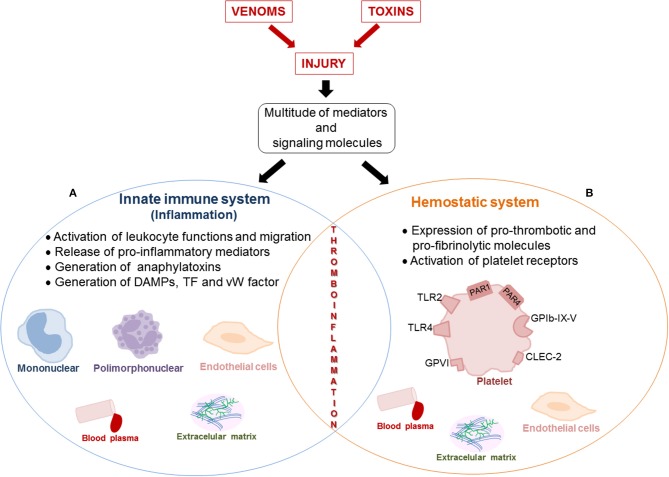
Schematic representation of the interplay between inflammation and alteration of the hemostatic system induced by venom and toxins that can favor thromboinflammation. The viperid snake venoms- and toxins-induced tissue injury trigger defense mechanisms in the victims, characterized by inflammation and hemostatic responses. Release of a multitude of mediators and signaling molecules that simultaneously act in the innate immune and hemostasis systems occurs, suggesting an overlap between both systems during viper envenomation. This interplay between inflammation and hemostatic alterations can characterize thromboinflammation. **(A)** Inflammation induced by snake venoms and toxins is characterized by migration of leukocytes, activation of cells (leukocytes and endothelial cells), which produce and release local and systemic pro-inflammatory mediators (IL-1β, IL-6, IL-8, TNF-α, MIP-1α, NO, histamine, serotonin, PAF, bradykinin, PGE_2_, TXA_2_, LTB_4_, and RANTES), generation of anaphylatoxins (C3 and C5) in blood plasma and DAMPs from extracellular matrix and cellular lysis. **(B)** Venoms and toxins induce hemostatic alterations, including platelet activation and aggregation, thrombin generation and suppression of the fibrinolytic system. Pro-coagulant toxins (prothrombin activators, thrombin-like enzymes, factor X and V activators), activate the expression of pro-thrombotic and pro-fibrinolytic molecules (vWfactor, tissue factor, fibrinogen, C5, and C3), which Interact with surface receptors (PAR-1, PAR-4, GPIb-IX-V, GPVI, TLR2, and TLR4) on platelets. Moreover, the activation of platelets by SV-CLRPs occurs in a non-enzymatic manner by interaction of toxins with the receptor CLEC-2. Several factors and signaling molecules of hemostatic system interfere with innate immune system, resulting in the amplification of inflammatory process and vice-versa.

The inflammatory response is closely associated with the onset of local and systemic toxicity induced by viperid venoms and their toxins. To date, the mechanisms underlying inflammation and blood coagulation induced by *Viperidae* venoms have been viewed as distinct processes. However, studies on the mechanisms related to inflammation and hemostatic alterations induced by viperid envenomation have evidenced participation of a multitude of mediators and signaling molecules that can simultaneously act in the innate immune and hemostasis systems. Toxins involved in these effects have also been identified, and for some of them, molecular mechanisms of action have been revealed. Therefore, the available information strongly suggests that an overlap between inflammation and hemostasis alterations, regarded as thromboinflammation, may occur during envenomation by viperid snakes (Figure 1). In this context, pro-inflammatory cytokines, which increase in production during viper venom-induced inflammation, have been demonstrated to induce hemostatic alterations, including platelet activation and aggregation, endothelial activation, thrombin generation, and suppression of the fibrinolytic system in several pathological conditions. Reciprocally, components of the hemostatic system also interfere with the immune system, leading to inflammation and amplifying this process. Moreover, besides pro-inflammatory activities, some *Bothrops* sp. SVMPs have been described to activate the expression of pro-thrombotic and pro-fibrinolytic molecules by endothelial cells and leukocytes, thus enhancing the coagulopathy seen in viperid snakebites. Similarly, SV-CLRPs activate platelet receptors, which are involved in both platelet aggregation and inflammation cascades. Therefore, SVMPs and SV-CRLPs are venom components selective for the activation of platelet functions and thrombus formation and are potent inducers of inflammation. This additionally argues for development of thromboinflammation during viperid envenomation. However, despite the evidence presented herein, the potential interplay between inflammation and hemostatic alterations induced by viperid snake venoms has never been reported. Therefore, comprehensive studies on the crosstalk between disturbances of hemostasis and inflammatory processes displayed by viperid snake envenomation are stressed. The impact of thromboinflammation on the toxic effects induced by *Viperidae* venoms also requires further investigation. Finally, we believe that this new aspect on the research of snake venom activities may bring renewed understanding of the complex pathology triggered by viperid snake venoms and toxins and may allow the discovery of new therapeutic targets and procedures to confront envenomation mortality and morbidity.

## Author Contributions

CT, CF, EL, and AC-T contributed to conception and design of the review, wrote the first draft of the review, contributed to text revision, read and approved the submitted version.

### Conflict of Interest Statement

The authors declare that the research was conducted in the absence of any commercial or financial relationships that could be construed as a potential conflict of interest.
